# The management of anticoagulants in patients with atrial fibrillation and history of falls or risk of falls: protocol for a systematic review and meta-analysis

**DOI:** 10.1186/s13643-022-01937-0

**Published:** 2022-04-08

**Authors:** Thibaut Galvain, Ruaraidh Hill, Sarah Donegan, Paulo Lisboa, Gregory Y. H. Lip, Gabriela Czanner

**Affiliations:** 1 School of Computer Science and Mathematics, Liverpool John Moores University, Liverpool, UK; 2 Liverpool Reviews and Implementation Group, Department of Health Data Science, University of Liverpool and The Royal Liverpool and Broadgreen University Hospitals, Liverpool Health Partners, Liverpool, UK; 3 Department of Health Data Science, University of Liverpool, Liverpool, UK; 4 School of Computer Science and Mathematics, Liverpool John Moores University, Liverpool, UK; 5 Liverpool Centre for Cardiovascular Science, Liverpool Heart and Chest Hospital, Liverpool, UK; 6 School of Computer Science and Mathematics, Liverpool John Moores University, Liverpool, UK

**Keywords:** Systematic review, Meta-analysis, VKA, NOAC, Falls, Atrial fibrillation, Anticoagulant

## Abstract

**Background:**

Atrial fibrillation affects an estimated 33 million individuals worldwide and is a major cause of stroke, heart failure, and death. Anticoagulants substantially reduce the risk of stroke but are also associated with an increased risk of bleeding and especially intracranial hemorrhage which is the most concerning complication. Because of this, many patients are not offered anticoagulants, particularly patients at risk of falls or with a history of falls. It is unclear what anticoagulant treatment these patients should be offered. The Liverpool AF-Falls project aims to investigate this area, and this protocol for a systematic review and meta-analysis aims to define what is the most appropriate anticoagulant treatment option for the management of atrial fibrillation patients at risk of falls or with a history of falls.

**Methods:**

This systematic review and meta-analysis will include randomized and non-randomized studies evaluating the safety and efficacy of different anticoagulant treatments (vitamin K antagonist and non-vitamin K antagonist oral anti-coagulant). Bibliographic databases (Cochrane Central Register of Controlled Trials, CINAHL, ClinicalTrials.gov, Embase, MEDLINE, Scopus and Web of Science) will be searched according to a pre-specified search strategy. Titles, abstracts, and full texts will be assessed by two independent reviewers and disagreements resolved with a third independent reviewer. The Cochrane Risk of Bias tool 2 (RoB 2) will be used to assess the risk of bias in randomized trials, and the Risk Of Bias In Non-randomized Studies - of Interventions (ROBINS-I) tool will be used for non-randomized studies. A pairwise meta-analysis based on the fixed and random-effects models will be conducted. Publication bias will be evaluated with a funnel plot and Egger’s test. Heterogeneity will be assessed with the *I*^2^ statistic. If conditions for indirect comparison are met and sufficient data are available, a network meta-analysis will be conducted using frequentist and Bayesian methodologies.

**Discussion:**

This review will be the first to summarize direct and indirect evidence on the safety and efficacy of anticoagulant treatments in atrial fibrillation patients at risk of falls or with a history of falls. The findings will be important to patients, clinicians, and health policy-makers to inform best practices in the use of these treatments.

**Systematic review registration:**

PROSPERO registry number: CRD42020201086

**Supplementary Information:**

The online version contains supplementary material available at 10.1186/s13643-022-01937-0.

## Background

Atrial fibrillation (AF) is the most common sustained cardiac arrhythmia [1] and is a major cause of stroke, heart failure, and death [2]. Stroke is the second most common cause of death, and it is a major cause of disability [3]. AF patients have a yearly risk of stroke of 5%, and this risk is increased in the presence of certain risk factors, including left ventricular dysfunction, hypertension, a history of stroke, and increasing age [4].

Treatment with oral anticoagulants substantially reduces the risk of stroke but are also associated with an increased risk of bleeding and especially intracranial hemorrhages which are the most concerning complication [5, 6]. Because of that, many patients do not receive anticoagulants, and particularly patients at risk of falls or with a history of falls [7]. For instance, in a French cohort of older people resident in nursing homes with AF and at high risk of stroke, less than 50% (541/1085) received anticoagulant treatment. Recurrent falls (47%), cognitive impairment (22.6%), and advanced age (16.4%) were the main reasons for not prescribing anticoagulants [8]. However, Donzé et al. showed that patients with oral anticoagulants at high risk of falls (*n*=308) did not have a significantly increased risk of major bleeds than patients at low risk of falls (*n*=207) (hazard ratio [HR] 1.09; 95% confidence interval, 0.54–2.21) [9]. Additionally, in elderly patients with AF, the considerable stroke risk without oral anticoagulant (OAC) often exceeds the bleeding risk on OAC [10].

There are two classes of anticoagulant treatments: (i) vitamin K antagonist (VKA) and (ii) non-vitamin K antagonist oral anticoagulant (NOAC). Anticoagulant treatments are recommended in most patients at risk of strokes except in those with low risk of strokes. Alongside these two classes, antiplatelet agents are also prescribed despite not being recommended for stroke prevention in AF patients, regardless of stroke risks [11]. In a recent meta-analysis in older AF patients, NOACs were associated with superior efficacy in preventing stroke/systemic embolism (HR 0.83, 95% CI 0.74–0.94) and non-inferiority safety for major bleeding (HR 0.93, 95% CI 0.86–1.01), intracranial bleeding (HR 0.58, 95% CI 0.50–0.67), and gastrointestinal bleeding (HR 1.17, 95% CI 0.99–1.38) compared to VKAs [12]. Due to the perceived risks of bleeding, many patients are not offered anticoagulants, particularly patients at risk of falls or with a history of falls. Thus, it is unclear what anticoagulant treatment these patients should be offered, and the Liverpool AF-Falls project aims to investigate this area.

This protocol for a systematic review and meta-analysis aims to define what is the most appropriate anticoagulant treatment option for the management of AF patients at risk of falls or with a history of falls.

### Research question and objectives

This review will explore the following research question: What is the most appropriate anticoagulant treatment strategy for AF patients at risk of falls or with a history of falls?

The objective of this systematic review and meta-analysis is to determine the safety and efficacy of different anticoagulant treatments for AF patients at risk of falls or with a history of falls.

## Methods

The protocol has been registered in the International Prospective Register of Systematic Reviews (PROSPERO) database (CRD42020201086). The methodology used for this systematic review follows the recommendations of the Cochrane Handbook for Systematic Reviews of Interventions [13]. The protocol is reported according to the Preferred Reporting Items of Systematic Reviews and Meta-Analysis for Protocols (PRISMA-P). The PRISMA-P checklist is available as an additional file (see Additional file 1). The review will be reported according to PRISMA 2020 [14], and PRISMA-NMA if a network meta-analysis is conducted [15].

### Eligibility criteria

#### Study designs

Randomized controlled trials (RCT) (including post hoc and ancillary analysis), quasi-randomized studies, and observational (prospective, retrospective, case-control and cohort studies) studies will be included. Non-randomized studies will be included to provide evidence of the effect over the long term that is incompletely addressed in randomized trials and for which only a small number of randomized trials is available. Animal studies, editorials, letters, case reports, reviews, case series, eminence-based opinions, and conference abstracts will be excluded. Non-standard RCT designs, such as cluster-randomized trials and crossover trials, will be excluded. Systematic reviews of interventions will be excluded, but included studies from relevant systematic reviews will be assessed for inclusion.

#### Types of participants

We will include studies of adults (age 18 or older) patients with any forms of nonvalvular AF (paroxysmal, persistent or permanent) with a history of falls or that are at risk of falls. Patients were defined as at risk of falls if they had one of these criteria [16]:

- Prior history of falls
- Lower extremity weakness
- Poor balance
- Cognitive impairment
- Vision and/or hearing impairment
- Orthostatic hypotension
- Use of psychotropic, or antihistaminic, or anticholinergic, or antihypertensive drugs
- Severe arthritis
- Dizziness.

Studies including patients receiving ablation, cardioversion or left-atrial appendage closure will be excluded as they represent relatively small and special subsets of patients.

#### Interventions and comparators

Eligible studies will include the following intervention: direct oral anticoagulants or non-vitamin K antagonist oral anticoagulants. Comparators will be vitamin K antagonists. Studies where patients receive combinations of treatments will be excluded.

#### Types of outcome measures

The primary efficacy outcome will be the composite of ischemic stroke and/or systemic embolism (an acute vascular occlusion of an extremity or organ). The primary safety outcome will be major bleeding (defined based on the International Society on Thrombosis & Haemostasis for major bleeding in non-surgical patients [17]). This definition of major bleeding includes (i) fatal bleeding and/or (ii) symptomatic bleeding in a critical area or organ, such as intracranial, intraspinal, intraocular, retroperitoneal, intra-articular or pericardial, or intramuscular with compartment syndrome, and/or (iii) bleeding causing a fall in hemoglobin level of 20 g/L−1 (1.24 mmol/L−1) or more, or leading to transfusion of two or more units of whole blood or red cells.

Secondary outcomes will include the following:

- Intracranial hemorrhage (Including all intracerebral, subdural, epidural, subarachnoid hemorrhage and hemorrhagic stroke)
- Gastrointestinal bleeding
- Clinically relevant non-major bleeding (defined based on International Society on Thrombosis & Haemostasis for major bleeding in non-surgical patients [18])
- Myocardial infarction
- Ischemic stroke
- Systemic embolism
- Hemorrhagic stroke
- Cardiovascular mortality
- All-cause mortality

### Search methods for identification of studies

#### Database searches

The following bibliographic databases will be searched: Cochrane Central Register of Controlled Trials (CENTRAL), CINAHL, Embase (via OVID); MEDLINE (via OVID), Scopus and Web of Science. We will also search the following trials register: the US National Institutes of Health Register (www.clinicaltrials.gov). Finally, we will double-check the reference lists of all the relevant studies and review the articles to identify additional relevant studies.

#### Search strategy

The search strategy for bibliographic databases was developed from the research question and implemented by a health sciences librarian with expertise in searches for systematic reviews. A combination of terms of medical subject headings (MeSH) and keywords will be used in the search strategy for MEDLINE through Ovid (see Additional file 2). For Embase, similar terms and search limits will be used. MeSH terms will be replaced with Emtree indexing terms and/or keywords, as appropriate. The search strategies for MEDLINE and Embase will be adapted for use in Scopus, Web of Science and the other bibliographic databases. The search results will be entered into the EndNote X8 reference management software for screening, once duplicate records are removed.

### Data collection and analysis

#### Selection of studies

Two stages of screening will be performed. Stage 1 screening will be conducted on the title and abstract of each citation to identify potentially eligible studies, applying the inclusion and exclusion criteria. The screening will be performed independently by two reviewers (TG and GC), with any questions resolved by consultation with a third reviewer (GL). All potentially eligible studies will be sought in full text for further screening and extraction. Stage 2, the full text will be evaluated independently by two reviewers (TG and GC) against the inclusion and exclusion criteria, with questions or discrepancies resolved by consultation with a third reviewer (GL). The reason for exclusion will be noted for all articles rejected at stage 2. Study authors will be contacted in case further information is needed to make a screening decision. A PRISMA flow diagram will be developed to record the study selection process [14].

#### Data extraction and management

A custom data extraction template will be designed for this review in Microsoft Excel and used for all eligible studies. Data will be extracted from each eligible study by one reviewer (TG) and cross-checked with the source article by a second reviewer (GC). Discrepancies and differences in interpretation will be resolved through discussion, and if necessary, by consultation with a third reviewer (PL or GL). Where insufficient data are presented, we will request additional information from the study authors by email. The following will be collected from each study: study characteristics (publication year, authors, title, study objectives and study outcomes), study population (such as age, gender, and diagnostic criteria), study design, intervention and control details, and outcomes including point estimates per group, treatment effect, confidence intervals and any p-value with associated statistical test. For RCTs, outcomes data will be sought on an intent-to-treat basis preferentially (results for all randomized patients, regardless of what treatment they received); however, where this information is not available, data will be extracted as reported by the authors. As-treated data may be analyzed if available. For observational studies, adjusted results will be preferred over non-adjusted, when available.

#### Assessment of risk of bias in included studies

The outcomes that will be assessed are listed in the section ‘types of outcome measures’. Outcomes are a time to event data, and the effect measure will be the hazard ratio.

In this systematic review, the risk of bias assessment of randomized controlled trials (RCTs) will be conducted with the Cochrane’s Risk of Bias 2 (RoB 2), where trials are scored as high, low, or ‘some concerns’ risk of bias within 5 domains: bias from the randomization process, bias due to deviations from intended interventions, bias due to missing outcome data, bias in the measurement of the outcome and bias in the selection of the reported result [13, 19]. The overall risk of bias will be reached using the signaling questions. The risk of bias assessment will be managed using the Excel tool to implement the ROB 2 [20].

The risk of bias in observational studies will be appraised with the Risk Of Bias In Non-randomized Studies - of Interventions I tool (ROBINS-I tool) [21]. Using this tool, studies are scored as low, moderate, serious or critical risk of bias. Confounding domains will include demographics, comorbidities, bleeding risk, stroke risk and concomitant treatments. Co-interventions will include anti-platelet agents.

The risk of bias will be independently evaluated by two reviewers (TG and GC) and will resolve any disagreements by discussion. Unresolved disagreements will be resolved through discussion with a third senior reviewer (GL). The effect of interest will be the effect of assignment for the ROB 2 and the ROBINS-I tools.

### Measures of treatment effect

#### Time to event data

Study outcomes are expected to be reported as a time to event data. We will record the number of participants per group and the hazard ratio between groups, with 95% CI and p value, as reported. If necessary, methods described by Tierney and al. could be used to retrieve the hazard ratio and its uncertainty [22, 23].

#### Dealing with missing data

In order to address loss to follow-up, a complete case analysis (based on a number of patients analyzed in included studies) will be conducted [24]. Additionally, we will request additional information from the study authors by email when data is missing.

#### Assessment of heterogeneity

Heterogeneity across studies will be assessed both by visual inspection of the forest plots and a formal statistical test, using Cochran *Q* test and *I*^2^ statistic [25]. An *I*^2^ up to 50% could suggest moderate-to-substantial heterogeneity, and more than 75% could suggest substantial heterogeneity. If high levels of heterogeneity are identified, i.e., *I*^2^ more than 50%, likely sources of heterogeneity will be further assessed using subgroup analysis (see “Subgroup analysis” section) and meta-regression, as data permit.

#### Assessment of risk of bias due to missing results

If more than 10 studies are included in the meta-analysis, selective outcome reporting and publication biases will be explored using a funnel plot to quantify the potential presence of publication bias. The asymmetry will be evaluated with Egger’s regression test [26].

#### Data synthesis

Summary statistics (study counts and patient numbers by key study characteristics) will be prepared from the set of studies meeting eligibility criteria, in order to assess the available evidence. Meta-analysis will be conducted based on the sufficient clinical homogeneity regarding participant characteristics, types of intervention and outcomes, and comparability between methods and ability to aggregate data [13]. Statistical heterogeneity as a consequence of clinical and/or methodological diversity will be evaluated using the *I*^2^ statistic. If heterogeneity is low or minor (*I*^2^ ≤ 25%), a fixed effect model will be used to pool the data; if heterogeneity is moderate-to-substantial (25% < I^2^ ≤ 75%), we will investigate the heterogeneity analysis and consider the usefulness of the random-effects model in order to take into account the clinical and methodological variation across studies; and if heterogeneity is substantial (*I*^2^ > 75%), a narrative synthesis will be conducted with tabular summaries of the extracted data and a forest plot without meta-analysis used to visualize results [25]. The decision to use a fixed- or random effect model will also be based on (i) an expectation of whether the intervention effects are truly identical and (ii) the funnel plot symmetry or asymmetry [13]. For the fixed effect model, the generic inverse variance method will be used. For the random-effects model, data will be pooled across studies using the DerSimonian and Laird model [27]. Results of the meta-analysis will be presented as pooled HRs with 95% CIs. A prediction interval will be generated to interpret a random-effects meta-analysis, which provides a predicted range for the true treatment effect in an individual study [28]. Meta-analyses will be conducted separately for RCTs and non-randomized studies. Individual study results and pooled estimates of treatment effect will be presented in tables and graphically using forest plots.

Following the pairwise meta-analysis, we will evaluate if it is appropriate to also conduct a network meta-analysis (NMA): (i) we will generate a network diagram to summarize the direct pairwise comparisons available; (ii) the homogeneity assumption of the evidence will be assessed by comparing potentially treatment effect-modifying, patient or trial characteristics, across trials that allocate the same two treatments and by generating a quantitative measure of heterogeneity [29]; (iii) the consistency assumption will be evaluated by comparing patient or trial characteristic across all trials in the NMA and by applying node-splitting to the NMA model [29]; and should we proceed with a network meta-analysis, we will conduct a Bayesian analysis for time to event outcomes. Estimated treatment effects will be reported as hazard ratios with 95% confidence intervals. Treatment rankings will be estimated with associated confidence intervals. The plan to conduct a network meta-analysis will be documented in a standalone protocol [30].

Statistical analyses will be performed in R using RStudio version 4.0.0 (meta, gemtc and metafor packages [31]).

#### Quality of evidence

Two reviewers (TG and GC) will assess the quality of evidence with the Grading of Recommendations Assessment, Development and Evaluation (GRADE) system which considers study design, risk of bias, inconsistency of results, indirectness, imprecision and other factors [32, 33]. Disagreements will be resolved by a third review author (PL or GL). Each study will be scored from very low, low, moderate to high quality of evidence using GRADEproGDT. Incorporating non-randomized studies evaluated with ROBINS-I in GRADE assessment will follow GRADE Guidelines 18 [34]. Summary of the evidence will be presented in a 'Summary of findings' table. This will provide key information about the best estimate of the magnitude of effect, in relative terms and as absolute differences [32, 35]. The following outcomes will be included in the summary of findings table:

- Ischemic stroke and/or systemic embolism
- Ischemic stroke
- Hemorrhagic stroke
- Major bleeding
- Intracranial hemorrhage
- All-cause mortality
- Cardiovascular mortality

#### Risk of bias between studies

If possible, subgroup analysis and meta-regression will be carried out to identify heterogeneity sources. The following variables will be investigated:

- Studies including patients at risk of falls (without a history of falls) compared to studies including patients with a history of falls
- Stratifying studies according to stroke risk evaluated with CHA_2_DS_2_-VASc score (CHADS_2_ in older studies)
- Stratifying studies according to bleeding risk assessed with the HAS-BLED score as patients may have different safety outcomes

Sensitivity analysis will be conducted to investigate the robustness of our findings. The analysis will be repeated including only studies identified as low risk of bias or according to the study design (randomized clinical trials and non-randomized studies). In case of inconsistency, the study characteristics will be further reviewed.

## Discussion

This systematic review and meta-analysis will synthesize the available evidence of the safety and efficacy of anticoagulant treatment used in patients with atrial fibrillation and at risk of falls or with a history of falls. Additionally, if the network meta-analysis is feasible, this will add even more value and benefits to evidence-informed practice and patient outcomes. Despite AF guidelines, stroke prevention through anticoagulation treatment for patients with atrial fibrillation and at risk of falls or with a history of falls is still subject to wide variation among clinicians. These patients might not receive anticoagulant treatment due to a perceived higher risk of bleeding complications. These risks have been assessed, but conclusive data regarding the risk-benefit trade-off are elusive. Additionally, it remains unclear what anticoagulant treatment (if any) these patients would benefit the most from. To our knowledge, this is the first systematic review on this topic and this work would provide clinicians and policy makers with information on which to make evidence-based recommendations. The study results will be submitted for publication in a peer-reviewed journal as well as a summary disseminated to the public in an accessible format.

## Supplementary Information

**Additional file 1.** PRISMA-P Checklist. Checklist for use with systematic review protocol submissions.

**Additional file 2.** Search strategy syntax for MEDLINE through Ovid. Detailed search strategy syntax used for MEDLINE through Ovid.

## Data Availability

Data sharing is not applicable to this article as no datasets were generated or analyzed during the current protocol. Results will be reported according to PRISMA 2020. The dataset that will be generated and analyzed during the current study will be available from the corresponding author on reasonable request.

## Abbreviations

AF: Atrial fibrillation
OAC: Oral anticoagulant
VKA: Vitamin K antagonist
NOAC: Non-vitamin K antagonist oral anticoagulant
HR: Hazard ratio
CI: Confidence interval
PRISMA-P: Preferred Reporting Items of Systematic Reviews and Meta-Analysis for Protocols RCT Randomized controlled trial
MeSH: Medical Subject Headings
ROBINS-I tool: Risk Of Bias In Non-randomized Studies - of Interventions
NMA: Network meta-analysis
