# Supplementary material for: The management of anticoagulants in patients with atrial fibrillation and history of falls or risk of falls: protocol for a systematic review and meta-analysis
Source: Syst Rev. 2022 Apr 8;11:63. doi: 10.1186/s13643-022-01937-0 (PMC8991693; doi:10.1186/s13643-022-01937-0)
Supplement: Supplementary file 2 — Additional file 2. Search strategy syntax for MEDLINE through Ovid. Detailed search strategy syntax used for MEDLINE through Ovid. [file 13643_2022_1937_MOESM2_ESM.docx]

# ****Additional file 2:**** Search strategy syntax for MEDLINE through Ovid

| 1 | exp *Atrial Fibrillation/ |
| --- | --- |
| 2 | atrial fibrillation.mp. |
| 3 | 1 or 2 |
| 4 | exp *Anticoagulants/ |
| 5 | exp *Platelet Aggregation Inhibitors/ |
| 6 | VKA.mp. |
| 7 | exp *Warfarin/ |
| 8 | exp *Acenocoumarol/ |
| 9 | exp *Dicumarol/ |
| 10 | exp *4-Hydroxycoumarins/ |
| 11 | exp *Phenindione/ |
| 12 | exp *Phenprocoumon/ |
| 13 | Phenprocoumon.mp. |
| 14 | Acenocoumarol.mp. |
| 15 | exp *Ethyl Biscoumacetate/ |
| 16 | Ethyl biscoumacetate.mp. |
| 17 | Fluindione.mp. |
| 18 | Clorindione.mp. |
| 19 | Warfarin.mp. |
| 20 | Dicumarol.mp. |
| 21 | 4-Hydroxycoumarins.mp. |
| 22 | Anticoagulant.mp. |
| 23 | Phenindione.mp. |
| 24 | antithrombo*.mp. |
| 25 | exp *Aspirin/ |
| 26 | Acetylsalicylic acid.mp. |
| 27 | exp *Clopidogrel/ |
| 28 | Clopidogrel.mp. |
| 29 | exp *Dipyridamole/ |
| 30 | Dipyridamole.mp. |
| 31 | Ticlopidine/ |
| 32 | Ticlopidine.mp. |
| 33 | exp *Prasugrel Hydrochloride/ |
| 34 | Prasugrel.mp. |
| 35 | exp *Dabigatran/ |
| 36 | exp *Factor Xa Inhibitors/ |
| 37 | Factor Xa Inhibitors.mp. |
| 38 | Dabigatran.mp. |
| 39 | apixaban.mp. |
| 40 | exp *Rivaroxaban/ |
| 41 | Rivaroxaban.mp. |
| 42 | edoxaban.mp. |
| 43 | 4 or 5 or 6 or 7 or 8 or 9 or 10 or 11 or 12 or 13 or 14 or 15 or 16 or 17 or 18 or 19 or 20 or 21 or 22 or 23 or 24 or 25 or 26 or 27 or 28 or 29 or 30 or 31 or 32 or 33 or 34 or 35 or 36 or 37 or 38 or 39 or 40 or 41 or 42 |
| 44 | exp *Accidental Falls/ |
| 45 | risk of fall*.mp. |
| 46 | fall*.mp. |
| 47 | lower extremity weakness.mp. |
| 48 | Paraparesis, Spastic/ |
| 49 | exp *Postural Balance/ |
| 50 | Postural Balance.mp. |
| 51 | exp *Cognitive Dysfunction/ |
| 52 | Cognitive Dysfunctions.mp. |
| 53 | exp *Hypotension, Orthostatic/ |
| 54 | poor balance.mp. |
| 55 | cognitive impairment.mp. |
| 56 | orthostatic hypotension.mp. |
| 57 | exp *Psychotropic Drugs/ |
| 58 | severe arthritis.mp. |
| 59 | exp *Dizziness/ |
| 60 | dizziness.mp. |
| 61 | exp *Arthritis/ |
| 62 | 44 or 45 or 46 or 47 or 48 or 49 or 50 or 51 or 52 or 53 or 54 or 55 or 56 or 57 or 58 or 59 or 60 or 61 |
| 63 | exp *Stroke/ |
| 64 | exp *Intracranial Hemorrhages/ |
| 65 | Intracranial haemorrhage.mp. |
| 66 | Major bleeding.mp. |
| 67 | exp *Mortality/ |
| 68 | mortality.mp. |
| 69 | systemic embolism.mp. |
| 70 | Ischaemic stroke.mp. |
| 71 | Haemorrhagic stroke.mp. |
| 72 | Cardiovascular mortality.mp. |
| 73 | Myocardial infarction.mp. or *Myocardial Infarction/ |
| 74 | exp Hemorrhage/ |
| 75 | *Gastrointestinal Hemorrhage/ or Gastrointestinal bleeding.mp. |
| 76 | bleeding.mp. |
| 77 | 63 or 64 or 65 or 66 or 67 or 68 or 69 or 70 or 71 or 72 or 73 or 74 or 75 or 76 |
| 78 | 3 and 43 and 62 and 77 |
| 79 | limit 78 to (english language and humans) |
| 80 | limit 79 to (adaptive clinical trial or clinical study or clinical trial, all or clinical trial or comparative study or controlled clinical trial or meta analysis or multicenter study or observational study or pragmatic clinical trial or randomized controlled trial or "review" or "systematic review") |

Where :

- “Exp” means that all of the narrower subject headings in the tree hierarchy will also be included which is the “Explode” option.
- “*” means that the “Focus” option has been selected. The "Focus" option tells the system that the retrieved citations should include the subject heading as a major focus of the articles.
- “.mp.” means a keyword multi-purpose search across several fields including title, abstract, original title, name of substance word, subject heading word, keyword heading word, protocol supplementary concept word, rare disease supplementary concept word, unique identifier.
- “/” means a subject heading search.
